# Selection for Social Signalling Drives the Evolution of Chameleon Colour Change

**DOI:** 10.1371/journal.pbio.0060025

**Published:** 2008-01-29

**Authors:** Devi Stuart-Fox, Adnan Moussalli

**Affiliations:** 1 School of Animal, Plant, and Environmental Sciences, University of the Witwatersrand, Johannesburg, South Africa; 2 Department of Zoology, The University of Melbourne, Melbourne, Australia; 3 School of Biological and Conservation Sciences, University of KwaZulu Natal, Pietermaritzburg, South Africa; 4 Department of Sciences, Museum Victoria, Melbourne, Australia; Emory University, United States of America

## Abstract

Rapid colour change is a remarkable natural phenomenon that has evolved in several vertebrate and invertebrate lineages. The two principal explanations for the evolution of this adaptive strategy are (1) natural selection for crypsis (camouflage) against a range of different backgrounds and (2) selection for conspicuous social signals that maximise detectability to conspecifics, yet minimise exposure to predators because they are only briefly displayed. Here we show that evolutionary shifts in capacity for colour change in southern African dwarf chameleons (Bradypodion spp.) are associated with increasingly conspicuous signals used in male contests and courtship. To the chameleon visual system, species showing the most dramatic colour change display social signals that contrast most against the environmental background and amongst adjacent body regions. We found no evidence for the crypsis hypothesis, a finding reinforced by visual models of how both chameleons and their avian predators perceive chameleon colour variation. Instead, our results suggest that selection for conspicuous social signals drives the evolution of colour change in this system, supporting the view that transitory display traits should be under strong selection for signal detectability.

## Author Summary

The ability to change colour has evolved in numerous vertebrate and invertebrate groups, the most well-known of which are chameleons and cephalopods (octopuses and their relatives). There is great variation among species, however, in the apparent capacity for colour change, ranging from limited changes in brightness to dramatic changes in hue. What drives the evolution of this remarkable strategy? We addressed this question by using a combination of field-based behavioural trials in which we quantified colour change, models of colour perception, and our knowledge of phylogenetic relationships for 21 distinct lineages of southern African dwarf chameleons. We show that evolutionary changes in the capacity for colour change are consistently associated with the use of social signals that are highly conspicuous to the visual system of chameleons. Moreover, capacity for colour change is unrelated to variation in the environmental backgrounds that chameleons must match in order to be camouflaged. Overall, our results suggest that the evolution of the ability to exhibit striking changes in colour evolved as a strategy to facilitate social signalling and not, as popularly believed, camouflage.

## Abstract 3

In dwarf chameleons, evolutionary shifts in the capacity for colour change are associated with increasingly conspicuous signals used in contests and courtship rather than by the need to match different backgrounds.

## Introduction

The ability to change colour in response to environmental stimuli has evolved in numerous vertebrate and invertebrate lineages including fish [1–4], amphibians [5,6], reptiles [7], crustaceans [8], and cephalopods [9,10]. In most lineages, colour change occurs over a period of minutes or hours and is primarily under hormonal control (e.g., [5,6,11]), whereas in some lineages, notably cephalopods and chameleons, chromatophores (pigment-containing cells or organs in the dermis) are under direct neural control, enabling the animals to respond extremely rapidly (within milliseconds or seconds) to changes in their natural or social environments [9,10,12]. For this reason, colour change in cephalopods and chameleons has featured in popular culture, myth and legend since first described in Aristotle's *Historia Animalium* [13]. In most lineages that have evolved colour change, however, the apparent capacity for colour change varies greatly. For instance, among the more than 150 species of the family Chamaeleonidae, colour change in some is primarily limited to shifts in brightness (e.g., shades of brown), while others show remarkable chromatic change, including striking combinations of blues, greens, oranges, yellows, and black [14]. Despite the animals' marked variation in the ability to change colour, processes driving the evolution of this adaptive strategy have never been examined.

Two primary processes may drive the evolution of colour change: (1) natural selection for the ability to camouflage against variety of backgrounds and (2) selection for conspicuous social signals. Like colourful hidden insect wings or plumage ornaments in some birds, colour change in a social context enables the use of signals that can be briefly exposed or flashed to intended receivers (usually conspecifics) but concealed from potential predators at other times. Because such “transitory signals” are only briefly exposed, they are expected to be under strong selection to maximise detectability to conspecifics [15,16], potentially explaining the evolution of dramatic colour change in some species. In many colour-changing lineages, colour change is known to facilitate both crypsis [5,10,14,17] and social communication [1,3,17–23]. As colour generally serves more than one purpose in colour-changing species, evolutionary processes underlying the ability to change colour per se cannot be inferred from functional studies of particular colour patterns. To infer underlying evolutionary processes requires experimental studies on closely related populations or taxa, which differ in their ability to change colour, or comparative tests based on phylogeny.

We used a phylogenetic comparative approach to test whether selection for crypsis or selection for conspicuous social signals (or a combination of the two) drives the evolution of colour change. We measured coloration and colour change (via reflectance spectroradiometry) in field-based behavioural trials conducted on 21 lineages of southern African dwarf chameleon, Bradypodion spp. We examined signal detectability (conspicuousness) by quantifying the visual contrast between display colours and the background against which they are viewed (vegetation and adjacent body regions), based on dwarf chameleon retinal photoreceptor spectral sensitivities and a model of colour perception. Because birds are a major predator of chameleons, we also applied this model using avian photoreceptor spectral sensitivities to explore the role of natural selection.

**Figure 1:**
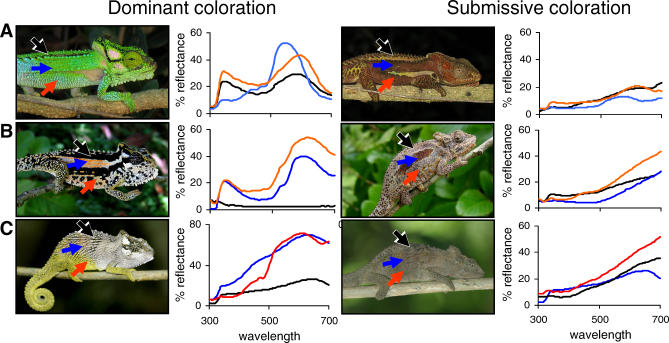
Displays and Reflectance Spectra for Three Species Spanning the Geographic Range of the Genus (A) B. damaranum from the south; (B) B. transvaalense from Woodbush in the north, and (C) B. caffrum from the central east. Panels from left to right: male in threat posture showing dominant coloration; associated reflectance spectra; male of the same population showing submissive coloration; associated reflectance spectra. Arrows indicate body regions from which measurements were taken (black = top flank, blue = mid-flank, red = bottom flank).

**Figure 2:**
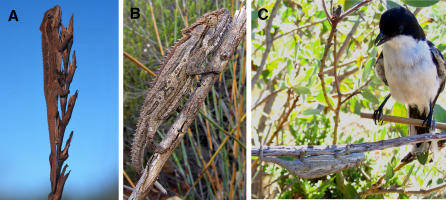
Examples of Camouflage and Antipredator Responses (A) B. taeniabronchum, a critically endangered species; (B) B. gutterale, and (C) B. atromontanum, showing typical antipredator behaviour (dorso-lateral flattening and flipping to the opposite side of the branch) in response to a model predator (stuffed fiscal shrike, Lanius collaris). All three are showing background matching whereby the animal's colour and pattern resembles a random sample of the background.

Dwarf chameleons are well suited to studying colour change because they vary greatly in the type and range of colours exhibited by each species. Males readily display to conspecifics and exhibit species-specific conspicuous colour patterns (Figure 1) both to signal dominance during aggressive interactions and to court females [24]. When a male loses a contest or is aggressively rejected by a female, it displays characteristic, species-specific submissive coloration (Figure 1). Females exhibit contrasting light and dark coloration during aggressive displays and, unlike some other chameleons [25], do not use colour patterns to signal reproductive status [20,24], instead displaying a subset of the colour variation apparent in males. Dwarf chameleons show greatest colour change during these interactions rather than in response to model predators [26] (Figure 2), therefore we measured colour change as the difference between male dominant and submissive coloration, which provides the best estimate of not only colour change within a social context but also capacity for colour change more generally.

We tested for an evolutionary correlation between the degree of colour change and multiple predictor variables, which were chosen based on expectations of the crypsis and social signalling hypotheses. The crypsis hypothesis predicts a positive relationship between colour change and the variance in background coloration, which chameleons need to match in order to be cryptic. Unlike cephalopods, which use a variety of camouflage strategies such as disruptive camouflage [10], dwarf chameleons rely primarily on background matching [26] (Figure 2). Natural selection can also act on colour patterns through variation in habitat structure, which will influence signal transmission and thus the detectability of signals to both chameleons and their predators [15,16]. Accordingly, we also tested for a relationship between colour change and three quantitative measures of habitat structure, which varies among the 21 lineages, from grassland to dense rainforest [24]. The social signalling hypothesis predicts a positive relationship between colour change and signal conspicuousness, which we estimate as the visual contrast between display colours and both the vegetation background and adjacent body regions, relative to the dwarf chameleon visual system. Colour change, however, may also be driven by sexual selection independent of signal detectability, because sexual selection may favour trait elaboration in directions that do not result in consistent changes in signal conspicuousness. We therefore include an independent measure of sexual selection as a potential predictor of colour change in our models. We used sexual dimorphism in the height of the ornamental casque, an intrasexually selected trait that predicts contest outcome in the Cape dwarf chameleon, Bradypodion pumilum [19].

Overall, our results support the predictions of the social signalling hypothesis but not the crypsis hypothesis, suggesting that the evolution of dramatic colour change in some chameleon species evolved as a strategy to facilitate social signalling. Our study demonstrates how quantifying signal detectability to animal sensory systems can inform our understanding of the evolution of signal diversity.

## Results/Discussion

We tested whether colour change is associated with variance in background coloration, three measures of habitat structure (principal components [PCs]), conspicuousness of dominance signals, and sexual dimorphism in casque height in a multivariate, phylogenetically controlled analysis. Because colour change may be constrained by body size, we included mean body size in all models.

Visual systems comprise two perceptual channels: the achromatic channel, which detects variation in light intensity (which we term “brightness” for simplicity) and the chromatic channel, which detects variation in the spectral composition of light, regardless of relative intensity [27]. Thus, we quantified the difference between two colours as the Euclidian distance between them in both brightness and chromatic space (see Materials and Methods), based on retinal photoreceptor spectral sensitivities of B. pumilum (E. R. Loew and L. J. Fleishman, unpublished data) (Figure S1A). It has been shown elsewhere for lizards that the more distant two patterns are in this space, the more distinct they are perceptually [28]. We assume that a signal's detectability is proportional to its conspicuousness (i.e., its contrast against the visual background).

**Figure 3:**
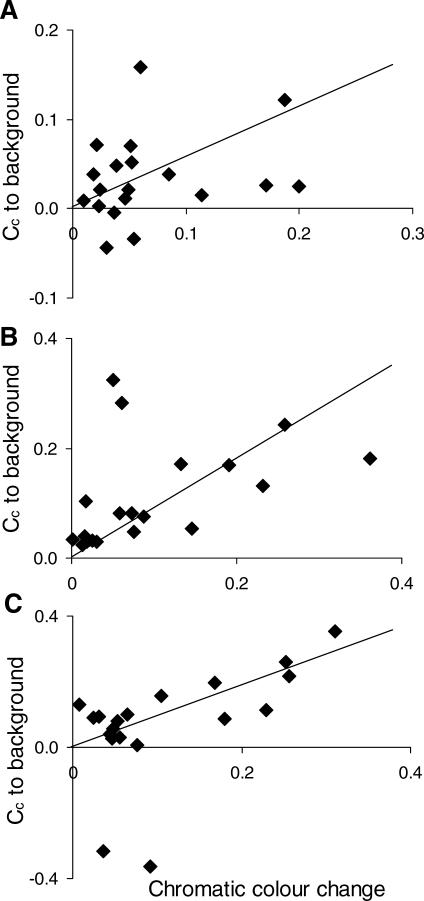
Relationship between Chromatic Colour Change and Conspicuousness of Dominant Colour Signals (A) Top flank (*r* ^2^ = 0.30, *p* = 0.008); (B) mid flank (*r* ^2^ = 0.54, *p* = 0.0001); (C) bottom flank (*r* ^2^ = 0.36, *p* = 0.003). Chromatic contrast against the vegetation background (*y*-axis) is denoted as C_C_ to background. Plots are regressions through the origin of Felsenstein's independent contrasts (FIC, positivized on the *x-*axis). For each variable, there are *N* – 1 contrasts, and one outlier was removed from each plot, resulting in 19 points (*r* ^2^ and *p-*values are for regressions with the outlier removed). The outlier in each case is the contrast between B. pumilum from Stellenbosch and B. pumilum from Vogelgat. These lineages are very closely related but differ greatly in both habitat (vegetation) and display coloration, resulting in large contrasts. Lines indicate regression slopes.

**Figure 4:**
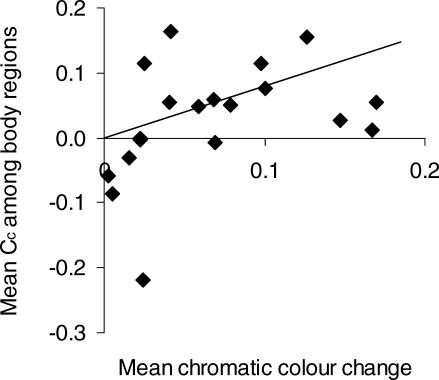
Relationship between Colour Change and Colour Contrast among Body Regions The plot shows the average colour change for all three body regions (top, mid-, and bottom flanks) regressed against the mean chromatic contrast (C_C_) of dominant signals among adjacent body regions (*r* ^2^ = 0.18, *p* = 0.04, with one outlier removed). The regression is based on Felsenstein's independent contrasts (FIC, positivized on the *x*-axis), regressed through the origin, with the regression slope indicated by the line. There are *N* – 1 contrasts and one outlier was removed, resulting in 19 points. As in Figure 3, the outlier is the contrast between B. pumilum from Stellenbosch and B. pumilum from Vogelgat.

We found that the species that show the greatest capacity for chromatic colour change have display signals that are highly conspicuous to conspecifics because of high visual contrast amongst body regions as well as with the vegetation background (Table 1). Increased capacity for chromatic change is accompanied by shifts to dominance signals that contrast more against the vegetation background, as perceived by the chameleon visual system for all three focal body regions (Figure 3). Additionally, mean chromatic change is positively associated with chromatic contrast among body regions (Figure 4).

**Table 1:**
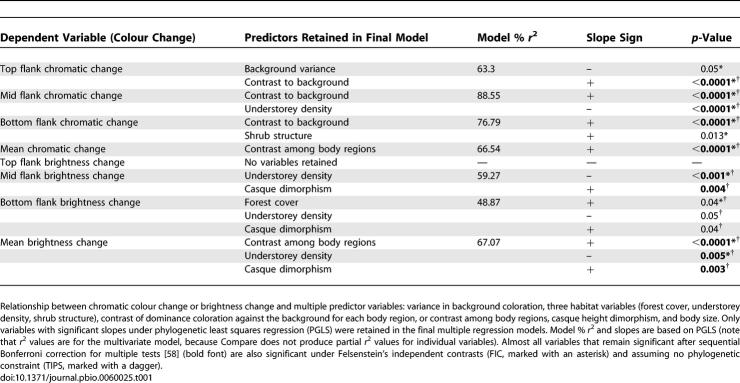
Predictors of Colour Change

Selection for conspicuous signals appears to influence the capacity for brightness change to a more limited extent. Brightness change is uncorrelated with brightness contrast against the background, although it is positively associated with brightness contrast amongst adjacent body regions (Table 1). However, mean brightness change and, particularly, brightness change of the mid-flank (the most conspicuous signal component in most species) are positively associated with casque height dimorphism (Table 1), indicating a role for sexual selection. Consistent with this view, the mid-flank in many dwarf chameleon species comprises a distinct colour badge, whose size predicts contest outcome in the Cape dwarf chameleon (B. pumilum) [19].

**Figure 5:**
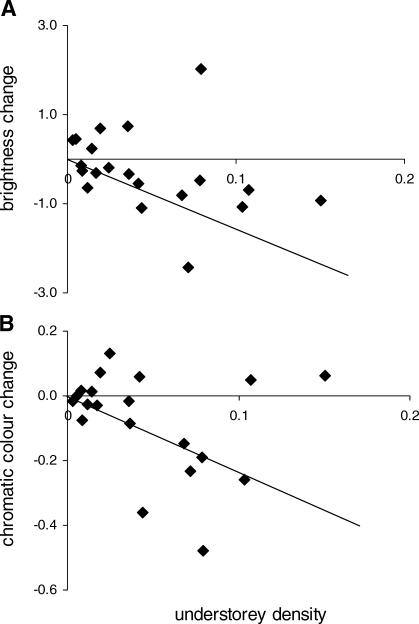
Relationship between Understorey Density and Colour Change Understorey density is principal component 2 from the principal components analysis of habitat structure (see Materials and Methods). (A) Change in brightness (*r* ^2^ = 0.14, *p* = 0.05) and (B) chromatic colour change (*r* ^2^ = 0.21, *p* = 0.02). The regressions are based on Felsenstein's independent contrasts (FIC, positivized on the *x*-axis), regressed through the origin, with the regression slope indicated by the line. There are *N* – 1 = 20 independent contrasts.

By comparison, we found no evidence that colour change in dwarf chameleons is associated with selection for crypsis. If capacity for colour change is driven by selection for the ability to match a variety of backgrounds, then colour change (in social or other contexts) should be positively associated with variance in background coloration—yet this is not the case (Table 1). Natural selection is nevertheless important. Species occupying habitats with a high density of stems or perches below 2 m, such as grasslands and heaths (high “understorey density”), show reduced colour change (both chromatic and brightness) of the mid-flank compared with species in habitats with an open understorey, such as mature rainforest (Figure 5). Natural selection can act on signals in two very different ways. The classical view is that natural selection favours reduced conspicuousness in some habitats due to variation in predation risk [29]. However, natural selection can also favour increased conspicuousness due to selection for signal efficacy (how well signals are transmitted and received in a given environment) [15]. To assess these alternatives, we modelled how both chameleons and their avian predators perceive dominant and submissive signals and tested whether signal conspicuousness to these different receivers is associated with variation in understorey density.

Dwarf chameleons have a range of potential avian predators [30], which include species having either of the two discrete categories of avian visual system, namely “ultraviolet-sensitive” (UVS, e.g., trogons and shrikes) (Figure S1B) and ”violet-sensitive” (VS, e.g., raptors) (Figure S1C) [31,32]. The crypsis hypothesis predicts that reduced colour change in habitats with dense understorey should be due to reduced conspicuousness of dominance signals to predators such as birds. However, we found no relationship between conspicuousness of mid-flank dominance signals and understorey density; this is true for both the avian and chameleon visual systems (brightness contrast birds: UVS and VS *r* ^2^ < 0.01, *p* = 0.73; brightness contrast chameleons: *r* ^2^ < 0.01, *p* = 0.62; chromatic contrast birds: UVS *r* ^2^ = 0.05, *p* = 0.3; VS *r* ^2^ = 0.05, *p* = 0.32; chromatic contrast chameleons: *r* ^2^ = 0.04, *p* = 0.99). This is consistent with the view that selection for crypsis is not the primary driver of colour change evolution in this system. Instead, in habitats with dense understorey vegetation such as heaths and grasslands, mid-flank submissive signals tend to be more conspicuous in terms of both brightness (brightness contrast birds: *r* ^2^ = 0.21, *p* = 0.05; brightness contrast chameleons: *r* ^2^ = 0.22, *p* = 0.02) and colour (chromatic contrast birds: UVS *r* ^2^ = 0.29, *p* < 0.0001; VS *r* ^2^ = 0.29, *p* = 0.05; chromatic contrast chameleons: *r* ^2^ = 0.36, *p* = 0.001), resulting in reduced colour change. This suggests selection for greater conspicuousness of submissive signals against a visually complex background (i.e., increased signal efficacy of submissive signals). An alternative, although not mutually exclusive, explanation is that habitats with dense, structurally complex understoreys may afford greater cover, reducing the need for submissive signals to be cryptic to avian predators.

These findings have important implications for understanding the evolution of animal signals. That animal signals reflect a trade-off between sexual selection for conspicuous (or costly) traits, and predator-driven natural selection for crypsis [33] is axiomatic. However, sexual and natural selection can also be mutually reinforcing when both favour signals that maximise detectability to conspecifics within a given environment [15,34]. This is particularly likely for transitory signals that are briefly displayed to conspecifics while remaining concealed from predators most of the time, such as the conspicuous colour patterns exhibited by some chameleons during social interactions. Our results are consistent with the expectation that transitory social signals should be minimally constrained by natural selection for crypsis and should instead be driven by selection for signal detectability. Although to our knowledge, there are currently no empirical data comparing signal detectability of transitory and continuously visible signals, both experimental and comparative studies confirm that habitat-related differences in signal detectability, in conjunction with sexual selection, can drive divergence in signalling traits [35–40]. Our study not only corroborates the importance of selection for signal detectability but demonstrates how quantifying signal conspicuousness to different receivers can be used to gain insights into the evolution of signal diversity in animals (see also [41]).

Although colour clearly functions in crypsis in dwarf chameleons (Figure 2), the ability to match different backgrounds does not appear to be the primary selective force in the evolution of colour change. Not only was colour change unrelated to variance in backgrounds, but the negative association between colour change of the mid-flank and understorey density was not due to decreased conspicuousness of display signals to potential avian predators. Ideally, however, the role of natural selection for crypsis should be tested more directly using data on relative predation risk in different habitats and should include other types of predators, such as snakes. It is also possible that apparently conspicuous body patterns used in social interactions are simultaneously camouflaged due to disruptive coloration [42,43]. This seems unlikely, however, because it requires that contrasting colour patterns break up the body's outline [44], which is not generally apparent in male displays (although it may well apply to females [24]).

We have shown that among dwarf chameleons, variation in capacity for chromatic change, but not brightness change, appears to be driven primarily by selection for conspicuous signals used in social interactions. Consistent with this finding, all dwarf chameleons show capacity for substantial change in brightness in a variety of contexts including thermoregulation [14], female aggressive rejection displays [20,24], and camouflage [26], whereas marked chromatic change is most prevalent in social contexts. Within a broader phylogenetic context, capacity for limited colour change is widespread among agamids and iguanids [7], the sister families to chameleons [45], as well as among basal chameleon genera (e.g., *Rampholeon* and *Brookesia*) [46,47]. In these groups, colour change is principally a function of brightness and is generally associated with thermoregulation and camouflage [7,14]. We suggest that although colour change may have originally evolved to facilitate thermoregulation or crypsis, the subsequent evolution of the remarkable capacity for chromatic change in some lineages of chameleons evolved as a strategy to facilitate social signalling.

## Materials and Methods

### Study system and spectroradiometry.

We collected data for 21 populations of dwarf chameleon (genus *Bradypodion sensu stricto*) (Table S1), which include all 14 currently described species and seven morphologically distinct, genetically divergent lineages [24,48]. Details of reflectance (of chameleons and their backgrounds) and irradiance measurements, habitat structure, phylogenetic reconstruction, and visual modelling are presented elsewhere [24].

For each population, we staged interactions between adult males on a perch in their natural habitat and measured dominant and submissive coloration, using an SD2000 spectroradiometer and PX2 light source (Ocean Optics) with illumination at 45° relative to the surface and reflectance measured at the same angle (optic fibres in parallel). The light source and spectrometer were connected via a bifurcating fibre optic cable to the probe, which was mounted in a probe holder (Ocean Optics RPH-2) to ensure readings were taken at a constant distance from the surface (6 mm). All trials were done in the shade under fine conditions to minimise thermal effects on colour. Measurements were relative to an Ocean Optics WS-1 diffuse reflectance standard, and standardisation was repeated between each individual. As soon as the chameleon showed clear aggressive behaviours (head-shake, lateral display, chase) or submissive behaviours (flee, flip to the under side of the branch to avoid the dominant male) and associated colour change, we took reflectance measurements for three to five body regions (top, mid-, and bottom flank, and any other minor colour pattern element on the mid-flank) in random order. By contesting each male against several different opponents, we obtained both dominant and submissive coloration for approximately five individuals per population (mean 5.5 ± 2.6 SD) (Table S1), enabling us to derive measures of individual colour change (measured as the visual contrast between display and submissive coloration—see below). This measure of colour change is both indicative of the range of colour change in the population and ensures that colours measured are consistent, because they are associated with specific behavioural responses. An alternative measure of colour change would be the difference between a “normal” or “resting” state and dominant coloration. However, measuring coloration while chameleons are in their “normal” or “resting” state is unreliable, because coloration when placed on a perch may reflect individually variable responses to capture, handling, or a human observer, whereas responses during male contests are consistent and reliable.

For backgrounds, we took reflectance readings of the leaves, branches, grass, or vines on which chameleons were caught and in the nearby vicinity. We then took the median reflectance of the primary (most common) type of background for each population, which we used in visual models. Additional details of measurement of background coloration are presented elsewhere [24].

### Visual modelling.

To model display colours as they would be perceived by chameleons or their avian predators, we used data on the median reflectance of the primary background colour, side-welling irradiance, and visual pigment absorbance functions for the Cape dwarf chameleon, B. pumilum (E. R. Loew and L. J. Fleishman, unpublished data) (Figure S1A), or birds (average values for UVS and VS systems from [49]) (Figure S1B and S1C). All calculations use population-specific background reflectance and irradiance measures. Details regarding the maximum absorbance of each type of visual pigment and template used (porphyropsin or rhodopsin) as well as cutoff wavelengths of associated oil droplets for B. pumilum are given in [24]. Both chameleons and birds are tetrachromats with four single cones and a double cone containing long wavelength–sensitive (LWS) visual pigments.

We first averaged each reflectance spectrum (of chameleons and their backgrounds) over each 5-nm interval using a kernel smoothing function. Next, we derived receptor quantum catches *Q* for each cone type *i* using the equation:

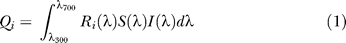
where λ represents wavelength, *R_i_* is the spectral sensitivity of cone type *i*, *S*(λ) is the reflectance of the colour patch, and *I*(λ) is the irradiance on the colour patch, integrated over the visible spectrum (300–700 nm; [49–51]). We used side-welling (parallel to the ground) rather than down-welling irradiance, as this is a more accurate measure of the light illuminating a chameleon's flank during lateral displays [24]. Visual pigment absorbance functions, *R_i_*, were corrected for filtering by ocular media and oil droplets and normalised to equal area to satisfy the assumption that all four cones are stimulated equally by white light [28,49].

Quantum catches of the four single cones were converted to relative quantum catches by dividing the quantum catch of each single cone by the sum of the quantum catches for all four cones:

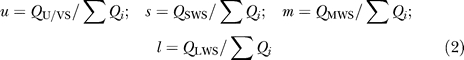
for the ultraviolet- or violet-sensitive (U/VS), short wavelength–sensitive (SWS), medium wavelength–sensitive (MWS), and long wavelength–sensitive (LWS) cones..

We calculated chromatic contrast (Δ_T_) of two colours (e.g., a display colour and vegetation or two adjacent body regions) as a function of the Euclidean distance in four dimensional (tetrachromatic) colour space based on the four single cone quantum catches [28]):

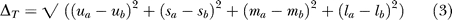
where (*u_a_*, *s_a_*, *m_a_*, *l_a_*) are the single cone relative quantum catches of colour *a* (e.g., the chameleon), and (*u_b_*, *s_b_*, *m_b_*, *l_b_*) are the relative quantum catches of colour *b* (e.g., a different body region or vegetation; [49]).

Brightness contrast (*C*_L_)was calculated as the difference between the double cone quantum catches (*Q*_D_) of the two colours divided by their sum [52]:

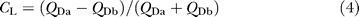



These measures of chromatic and brightness contrast assume that the greater the difference in the relative stimulation of the four single cones or the double cone, the more different they will appear to the receiver [28]. The measure of chromatic contrast makes several implicit assumptions about colour perception, the most important of which is that all four cones contribute equally to colour perception. In reality, however, colour perception is likely to be influenced by the relative proportion and distribution of cone types within the retina, which can vary even among closely related species, at least in birds [53]. Moreover, this measure ignores opponency mechanisms (comparison of outputs of different cone types) known to be important for colour discrimination [27]. Other, more complex models that do take these factors into account have been developed for birds [51]; however, these require additional data that are not available for chameleons and predict discrimination between similar colours, rather than how different two colours appear to a receiver. In the absence of information on how the chameleon retina and brain processes photoreceptor quantum catches, the simpler model we use is reasonable and accurately predicts signal detectability in the lizard Anolis cristatellus [28]. We used the same model for birds for consistency and to ensure that our results were not due to different model assumptions.

For each individual, we calculated the distance in colour or brightness space for the following scenarios: (1) between chameleon dominant and submissive colours as our measure of colour change; (2) between dominant colours and the vegetation background; (3) between dominant colours of adjacent body regions; and (4) between mid-flank dominant colours and the background and between mid-flank submissive colours and the background, to both bird and chameleon vision. For (1) and (2), we only used the top, middle, and bottom flanks, because these body regions were comparable between all 21 lineages. For (3), we calculated the contrast between each pair of adjacent body regions and took the mean of these pair-wise comparisons (i.e., we derived mean contrast among adjacent body regions for each individual).

### Variance in background coloration.

We measured background variance for use in comparative analyses (i.e., to test the crypsis hypothesis) as follows. First, we calculated the total brightness of each background as its integral over the visible spectrum (i.e., area under the curve), then we calculated the variance across the brightness of backgrounds for each population. For the chromatic variance, we first standardised background reflectance measures to have equal brightness, then calculated the variance at each 5-nm interval and took the average of these variances over the visible spectrum.

### Habitat structure.

Measures of habitat structure are derived from a principal components analysis (PCA) of nine variables that we measured for each population within five 10 × 10–m plots in which chameleons had been found. Full details regarding the habitat variables and the results of the PCA are presented elsewhere [24,54]. The first three principal components (PCs) explain 82% of the variation in habitat structure. PC1 (“forest cover”) is positively associated with canopy cover, canopy height, number of trees, vine cover, and shrub height and therefore differentiates more closed, forested habitats from more open habitats. PC2 (“understorey density”) is positively associated with perch density below two metres and differentiates habitats such as grasslands or heaths with a dense herb or shrub understorey from habitats with a sparse understorey. PC3 (“shrub structure”) is negatively associated with number of shrubs and trees and positively associated with shrub width.

### Phylogenetic comparative analyses.

For comparative analyses, we used phylogenetic generalized least squares (PGLS) [55] and Felsenstein's independent contrasts (FIC) [56] implemented in Compare 4.06 [57]. We used a molecular phylogeny [24,48,54] and ran all analyses on both the fully resolved tree and a tree with branches with less than 0.9 Bayesian posterior probability collapsed, i.e., treating these nodes as polytomies. Results were qualitatively the same for both; therefore we present only those for the fully resolved tree. The phylogeny and phylogenetic methods are given in [24] and [54]. Measures of sexual dimorphism in casque height, body size, and habitat structure are from [54]. We used sexual dimorphism in casque height as an index of sexual selection rather than the commonly used sexual dimorphism in body size, because dwarf chameleons show either no sexual size dimorphism (SSD) or reversed SSD (females larger than males) [54], and male body size does not predict contest outcome [19]. We used population means for each variable in comparative analyses.

## Supporting Information

Figure S1 Visual Pigment Relative Absorbances for the Cape Dwarf Chameleon (B. pumilum) and the Two Types of Avian Visual Systems (UVS and VS) (54 KB PPT)

Table S1 Sampling Localities and Sample Sizes for Measures of Both Dominant and Submissive Coloration (43 KB DOC)

## Abbreviations

UVS: ultraviolet-sensitive
VS: violet-sensitive
