# Supplementary material for: Selection for Social Signalling Drives the Evolution of Chameleon Colour Change
Source: PLoS Biol. 2008 Jan 29;6(1):e25. doi: 10.1371/journal.pbio.0060025 (PMC2214820; doi:10.1371/journal.pbio.0060025)
Supplement: Figure S1 — (54 KB PPT) [file pbio.0060025.sg001.ppt]

## Slide 1
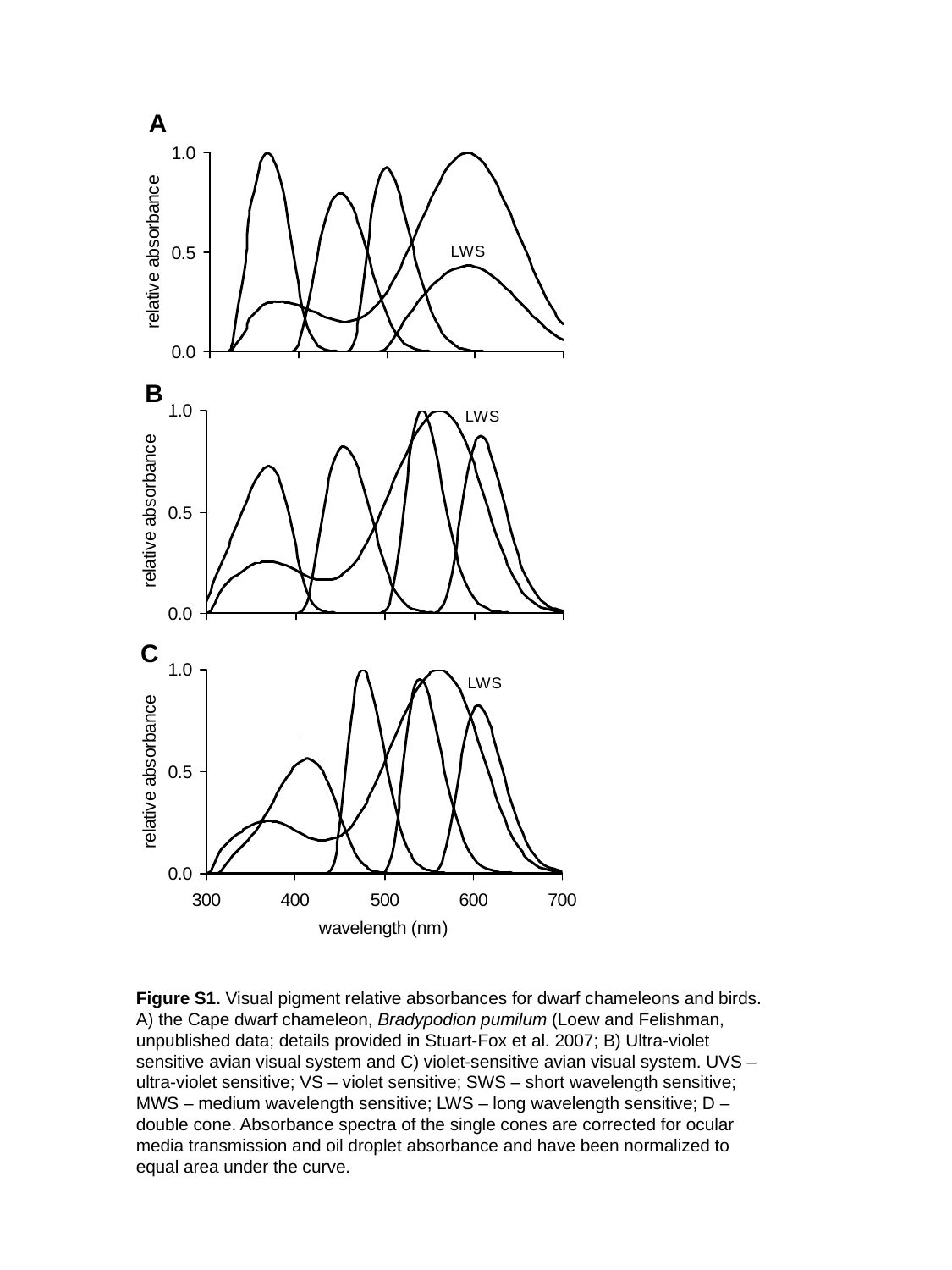

Figure S1. Visual pigment relative absorbances for dwarf chameleons and birds. A) the Cape dwarf chameleon, Bradypodion pumilum (Loew and Felishman, unpublished data; details provided in Stuart-Fox et al. 2007; B) Ultra-violet sensitive avian visual system and C) violet-sensitive avian visual system. UVS – ultra-violet sensitive; VS – violet sensitive; SWS – short wavelength sensitive; MWS – medium wavelength sensitive; LWS – long wavelength sensitive; D – double cone. Absorbance spectra of the single cones are corrected for ocular media transmission and oil droplet absorbance and have been normalized to equal area under the curve.
