# Supplementary material for: Selection for Social Signalling Drives the Evolution of Chameleon Colour Change
Source: PLoS Biol. 2008 Jan 29;6(1):e25. doi: 10.1371/journal.pbio.0060025 (PMC2214820; doi:10.1371/journal.pbio.0060025)
Supplement: Table S1 — (43 KB DOC) [file pbio.0060025.st001.doc]

**Supporting Information**

**Table S1.** Sampling localities and sample sizes for measures of both dominant and submissive coloration.

| Population | Locality | N |
| --- | --- | --- |
| *B. sp. 1.* | Swartberg Pass, WC | 7 |
| *B. sp. 2.* | Dhlinza Forest, Eshowe, KZN | 7 |
| *B. sp. 3.* | Ngome Forest Reserve, KZN | 3 |
| *B. caffrum* | Port St Johns, EC | 9 |
| *B. damaranum* | Between George and Knysna, WC | 7 |
| *B. dracomontanum* | Royal Natal National Park, KZN | 1 |
| *B. gutterale* | Anysberg NR, WC | 5 |
| *B. kentanicum* | Vicinity of Kentani, | 4 |
| *B. melanocephalum* | Kennethstainbank NR, Durban, KZN | 4 |
| *B. nemorale* | Nkandla Forest Reserve, KZN | 2 |
| *B. occidentale* | Paternoster, WC | 10 |
| *B. pumilum* | Stellenbosch, WC | 6 |
| *B. pumilum* | Vogelgat NR, WC | 2 |
| *B. setaroi* | St Lucia, KZN | 10 |
| *B. taeniabronchum* | Lady’s Slipper, EC | 4 |
| *B. thamnobates* | Bulwer, KZN | 4 |
| *B. transvaalense* | Barberton, Mpumalanga | 5 |
| *B. transvaalense* | Tullach Moor NR (Eland’s Valley), MP | 7 |
| *B. transvaalense* | Vicinity of Graskop, MP | 7 |
| *B. transvaalense* | Woodbush Forest, LP | 8 |
| *B. ventrale* | Vicinity of Grahamstown, EC | 4 |

Abbreviations for provinces: EC – Eastern Cape, WC – Western Cape, MP – Mpumalanga, LP – Limpopo, KZN – KwaZulu Natal. NR stands for Nature Reserve.
